# Antioxidant and Antibacterial Activities Evaluation, Phytochemical Characterisation of Rhizome from *Angiopteris helferiana* and Barks from *Saurauia fasciculata* in Nepal

**DOI:** 10.1155/2024/1119165

**Published:** 2024-06-12

**Authors:** Ram Kishor Yadav, Akriti Dhakal, Kalpana Timilsina, Priyanka Shrestha, Sandesh Poudel, Sindhu KC, Prabhat Kumar Jha, Rekha Bhandari, Khem Raj Joshi

**Affiliations:** School of Health and Allied Sciences, Pokhara University, Pokhara, Nepal

## Abstract

Ethnomedicinally, more than 2000 plants were found to be used in Nepal. Among them, the red colored rhizome of *Angiopteris helferiana* and the bark of *Saurauia fasciculata* have been used widely to treat muscle fatigue, bone pain, fever, postpartum hemorrhage, and thirst by healers in Kaski and Tanahun districts, Nepal. However, scientific evidence towards their traditional uses is lacking till December, 2023. Therefore, we report the phytochemicals, total phenolic content (TPC), total flavonoid content (TFC), total carbohydrate content (TCC), antioxidant and antibacterial activities of *A. helferiana* and *S. fasciculata* extracts. Phytochemical analysis indicated that *A. helferiana* and *S. fasciculata* extracts were potential sources of chemicals such as phenols, flavonoids, tannins, terpenoids, saponins, and carbohydrates. The TPC, TFC, and TCC of extracts were determined by using an ultraviolet visible spectrophotometer. Among the extracts tested, *A. helferiana* extracts showed the highest phenolic and carbohydrate contents of 208.33 ± 12.96 mg of gallic acid equivalent/g and 564.16 ± 2.92 mg of D-glucose equivalent/g of dry extract, respectively. Similarly, *S. fasciculata* revealed the highest flavonoid content of 30.35 ± 0.1 mg quercetin equivalent/g of dry extract. The extract of *A. helferiana* and *S. fasciculata* exhibited potent antioxidant activity by scavenging 2,2-diphenyl-1-picrylhydrazyl radicals with an IC_50_ of 25.9 *µ*g/ml and 31.07 *µ*g/ml, respectively. The antibacterial activity of the *A. helferiana* and *S. fasciculata* extract against *Staphylococcus aureus, Pseudomonas aeruginosa*, and *Escherichia coli* was determined using an agar-well diffusion protocol that revealed the potential antibacterial activity of *A. helferiana* against *E. coli*. The present study will help validate the traditional uses of *A. helferiana* rhizomes and *S. fasciculata* barks as a healing medicine and inspire the researcher towards further research, development, and formulation.

## 1. Introduction

Nepal's diverse altitude, topography, and climate contribute to its significant floral biodiversity with 6500 flowering plants and fern species [1]. The people of Nepal have a deep respect and belief in medicinal plants. Approximately 2000 medicinal and aromatic plants are traditionally used for their healing potential [2]. Among them, *Angiopteris helferiana* and *Saurauia fasciculata* are well-recognized medicinal plants.

*Angiopteris helferiana* C. Presl. belongs to the Marattiaceae family and is characterised as a gigantic fleshy fern with a massive terrestrial rhizome. It is distributed at an altitude of 900–1400 meters in moist forests throughout Southeast Asia, mostly in Nepal, India, China, and Sri Lanka [3]. Rhizome of *A. helferiana* is traditionally prescribed to cure muscle and bone pain/fatigue in Nepal [3], scabies in India [4], and dysentery, infection, scabies, and muscle pain in Bangladesh [5].

To the knowledge of ethnomedicinal healers from the Kaski and Tanahun districts of Nepal, Gai-Khurey (*A. helferiana*) exists in two local cultivars which are locally recognized by its rhizome color as Rato Gai-Khurey (red colored rhizome) and believed to be more ethnomedicinally potent in muscle and bone pain/fatigue than Seto Gai-Khurey (light yellow colored rhizome). Previous studies conducted on *A. helferiana* (light yellow rhizome) have reported antioxidant, anti-inflammatory, antiobesity, and antidiabetic activities. Furthermore, few lactones such as (−)-epi-osmundalactone and angiopteroside were isolated from the same cultivars [4]. However, *A. helferiana* with red color rhizome remains unexplored until December 2023.

**Figure 1:**
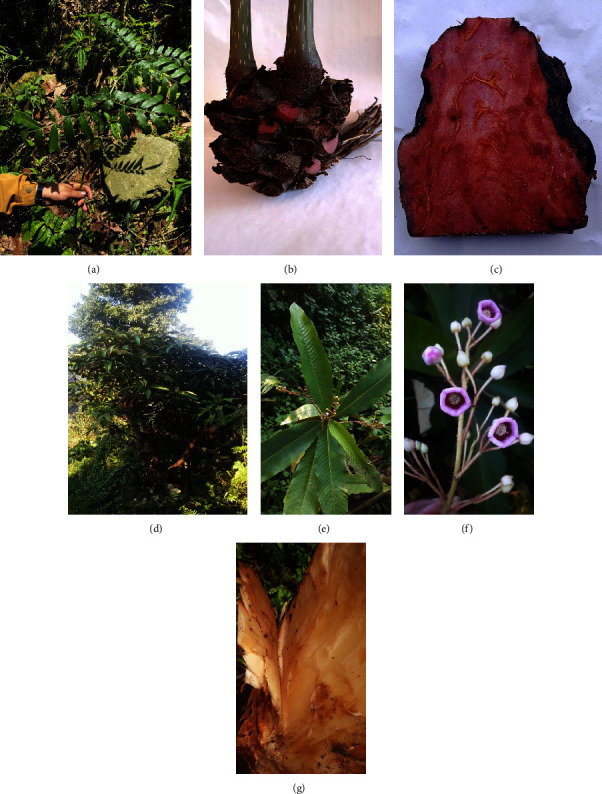
Photographs of *A. helferiana*: (a) whole plant; (b) rhizome; (c) cross-section of rhizome; and *S. fasciculata*: (d) whole plant; (e) leaves; (f) inflorescence; (g) bark captured from its natural habitat in May, 2023.

*Saurauia fasciculata* Wall. (Syn *Ternstroemia fasciculata*), a member of the Actinidiaceae family, is a 3–6 m tree with 10–25 by 3–8 cm elliptic-oblong to elliptic-lanceolate leaves, 5–9 cm long inflorescence crowded behind the tip of branchlets, and 6.5–9 by 5–5.5 mm flower [6] (Figure 1). In Nepal, *S. fasciculata* is locally recognized as Goban and its bark juice is given orally in fever, postpartum hemorrhage, and thirst [7]. Previous studies carried out on various other species of the genus Saurauia revealed several biological activities such as antioxidant, antidiabetic, analgesic, wound healing, and antihyperlipidemic [8]. However, no studies have been carried out on the bark of *S. fasciculata* up to December, 2023.

The present study therefore, aims to analyze phytochemicals and evaluate antioxidant and antibacterial activity of extracts from the red-colored rhizome of *A. helferiana* and the bark of *S. fasciculata.*

## 2. Materials and Methods

### 2.1. Materials

#### 2.1.1. Chemicals and Bacterial Strains

Ascorbic acid, chloroform, gallic acid, precoated silica gel 60 F_254_, and methanol (Merck, India), anhydrous ferric chloride, D-glucose, silica gel cc (230–400 mesh), and sulfuric acid (Thermo Fisher Scientific, India), Folin–Ciocalteu reagent (SDFCL, India), 2,2-diphenyl-1-picrylhydrazyl (DPPH), Muller–Hinton Agar, and Quercetin dihydrate (HiMedia, India) were used for the experiments. *Staphylococcus aureus*, *Pseudomonas aeruginosa*, and *Escherichia coli* were obtained from Manipal Teaching Hospital, Pokhara, Nepal.

#### 2.1.2. Plant Collection and Authentication

The fresh fronds and rhizome of *A. helferiana* were collected in May 2023 from the Vyas municipality, Tanahun district, Nepal, and the bark of *S. fasciculata* was collected in May 2023 from Hemja, Kaski district, Nepal. The authenticity of the plant specimen (31/02/2080/Herbarium specimen Nos 2 and 5) was confirmed at the National Herbarium and Plant Laboratories, Godawari-3, Lalitpur, Nepal, by referring to the deposited plant specimen.

### 2.2. Extraction Procedures

The fresh rhizomes of *A. helferiana* and the barks of *S. fasciculata* were cleaned with tap water, reduced in size with a plant cutter, and then shade dried for one week to eliminate moisture. Using the methodology of [9, 10], extraction was carried out.

#### 2.2.1. Preparation of Extracts for Qualitative Phytochemical Analysis

The red *A. helferiana* rhizomes (30 g) and the barks of *S. fasciculata* (30 g) were separately subjected to extraction using 70% methanol as a solvent in a ratio of 1 : 10 w/v at room temperature (24°C) with frequent agitation for 24 hours. The liquid crude extract was filtered using a cotton plug and used for qualitative phytochemical analysis.

#### 2.2.2. Preparation of Extracts to Estimate Extractive Yield, TLC Profiling, Quantitative Phytochemical Analysis, Antioxidant Activity, and Antibacterial Activity

The *A. helferiana* rhizomes (100 g) and the barks of *S. fasciculata* (150 g) were separately subjected to successive extraction using 70% methanol as solvent in the ratio of 1 : 10 w/v at 55°C for 2 hours followed by room temperature (24°C) for 22 hours and finally 70% methanol at room temperature (24°C) for an additional 24 hours. The crude liquid extracts were filtered using a thick cotton plug and combined to evaporate under reduced pressure at 55°C using a rotary evaporator (Biobase RE-2000B, Germany). The concentrated extracts obtained after evaporation were poured in a beaker, followed by complete drying in vacuum desiccators. The dried extracts were weighed and stored at 4°C in refrigerator until use. The extractive yield was calculated using the formula:

(1) $$\begin{array}{c} \%\text{ extractive yield}=\left(\frac{\text{weight of dry extract}}{\text{initial weight of dry sample}}\right)\times 100. \end{array}$$

### 2.3. Qualitative Phytochemical Analysis

Phytochemicals such as alkaloids, phenols, flavonoids, tannins, carbohydrates, saponins, terpenoids, anthraquinone, steroids, and proteins were analysed in the rhizome of *A. helferiana* and the bark of *S. fasciculata* according to the methodology of [11, 12]. All the reagents used for analysis were prepared in distilled water.

#### 2.3.1. Test for Phenols (FeCl_3_ Test)

A 2% of FeCl_3_ solution was prepared, and 2 ml of it was treated with 2 ml of crude liquid extract. Blue, green, or black suggested the presence of phenolic compounds.

#### 2.3.2. Test for Flavonoids

*(1) Alkaline Reagent Test*. Plant extracts (2 ml) were treated with 2 ml of 2% NaOH, followed by the addition of a few drops of aqueous HCL. Appearance of an intense yellow with NaOH, which changes to colorless upon addition of HCL, indicated the presence of flavonoids.

*(2) Shinoda Test*. A fragment of magnesium ribbon reacted with 2 ml of extract solution and a few drops of HCL. Appearance of red or pink after a few minutes suggested flavonoids.

*(3) Lead Acetate Test*. Crude liquid extract (2 ml) was mixed with a few drops of lead acetate solution; a yellow precipitate indicated flavonoids.

#### 2.3.3. Test for Tannins

*(1) *FeCl*_3_ Tests*. A 2% of FeCl_3_ solution was prepared, and 2 ml of it was treated with 2 ml of crude liquid extract. Blue, green or black suggested the presence of tannins.

*(2) Lead Acetate Test*. Crude liquid extract (2 ml) was mixed with a few drops of 10% lead acetate solution; a white-yellow precipitate indicated tannins.

*(3) Lime Water Test*. Lime water was prepared, and 2 ml of it was mixed with 2 ml of crude extract; precipitate formation suggested the presence of tannins.

*(4) Gelatin Tests*. Gelatin (0.5%) was prepared in 10% of NaCl, and 2 ml of it was treated with an equal volume of test extract; turbidity indicated the presence of tannins.

#### 2.3.4. Test for Saponin (Foam Test)

Test extract (2 ml) was shaken with 5 ml of distilled water for a few minutes in a closed test tube; the formation of stable foam confirmed the presence of saponin.

#### 2.3.5. Test for Alkaloids

*(1) Mayer's Reagent Test*. 2 ml of reagent was treated with 2 ml of liquid crude extract; a white-yellow precipitate suggests alkaloids.

*(2) Wagner's Reagent Test*. Crude extract (2 ml) was treated with a few drops of reagent; a brown precipitate indicated the presence of alkaloids.

*(3) Hager's Reagent Tests*. A few ml of extract solution was mixed with 1 ml of reagents. A creamy white precipitate indicated alkaloids.

#### 2.3.6. Test for Terpenoids and Steroids (Salkowski Test)

2 ml of concentrated H_2_SO_4_ was added to the mixture containing an equal volume of extract solution and CHCl_3_. After gently shaking, the appearance of a reddish-brown color in the layer of CHCl_3_ and green fluorescence in the layer of H_2_SO_4_ layer confirmed the presence of steroids or triterpenoids.

#### 2.3.7. Test for Carbohydrates

*(1) Fehling's test*. A mixture of test extract (2 ml) and Fehling's solution (2 ml) was heated for 10 min over a hot water bath; a precipitate with a brick red color confirmed the presence of reducing sugar.

*(2) Benedicts test*. A mixture containing test extract (2 ml) and Benedict's solution (2 ml) was heated for 2 min over a hot water bath; a precipitate with reddish-brown color indicated the presence of carbohydrates.

#### 2.3.8. Test for Protein (Ninhydrin Test)

A 0.2% of ninhydrin solution was prepared, and 1 ml of it was treated with an equal volume of extract solution and boiled for 5 min; a purple color suggested the presence of amino acids or proteins.

#### 2.3.9. Anthraquinone Test (Borntrager's Tests)

2 ml of extract was mixed with 2 ml of 10% NaOH solution. A red color developed. Afterwards, a small volume of 30% H_2_O_2_ solution was added and the mixture was heated at 60°C. HCl solution was then added, and the red color disappeared; finally, NaOH solution was added and the development of a red color indicated the presence of anthraquinones.

### 2.4. Quantitative Estimation of Phytochemicals

#### 2.4.1. Estimation of the Total Phenolic Content (TPC)

The TPC was estimated spectrophotometrically by implementing the Folin–Ciocalteu's reagent method adopted previously [13], with slight modifications. Briefly, 100 *μ*l of extract (1000 *µ*g/ml in distilled water), 6 ml of distilled water, and 0.5 ml of Folin–Ciocalteu phenol reagent (2 N) were homogenised in a test tube for 10 seconds using a Vortex mixer. Subsequently, after 5 minutes, 1.5 ml of sodium carbonate (7.5%) and 1.9 ml of distilled water were added, homogenised, and incubated for 2 hours in the dark. For the calibration curve, gallic acid (31.25 *µ*g/ml–500 *µ*g/ml) was considered a reference phenolic compound and treated similarly to the extract. At 750 nm, a single beam UV-VIS spectrophotometer (Agilent Cary 60, Malaysia) was used to read the absorbance of the solution. In the blank solution, extract (100 *μ*l) was replaced with water (100 *μ*l) and treated identically to extract. The mean absorbance of triplicates per sample was used, and TPC was expressed as milligrams of gallic acid equivalents (mg of GAE)/g of extract.

#### 2.4.2. Estimation of the Total Flavonoid Content (TFC)

The TFC was estimated by implementing the AlCl_3_ method adopted by [11], with slight modifications. Briefly, 2 ml of AlCl_3_ (2% in distilled water) and 2 ml of extract (100 *µ*g/ml of water) were mixed together and incubated at room temperature for 10 minutes. For the calibration curve, quercetin (1.25–100 *µ*g/ml) was considered as a reference flavonoid and treated in a similar way to the extract. At 415 nm, a single beam UV-VIS spectrophotometer was used to read the absorbance of the solution against a blank consisting of extract with distilled water. The mean absorbance of triplicates per sample was used to express TFC as milligrams of quercetin equivalent (mg of QE)/g of extract.

#### 2.4.3. Estimation of the Total Carbohydrate Content (TCC)

The TCC was estimated spectrophotometrically as described by [11], with slight modification. Briefly, 1 ml of plant extract (250 *µ*g/ml), 0.5 ml of phenol aqueous solution (5%), and 2.5 ml of concentrated H_2_SO_4_ were mixed in a test tube and incubated for 30 min. A calibration curve was produced based on D-glucose (12.5–200 *µ*g/ml) as a standard. At 490 nm, absorbance was measured against a blank consisting of distilled water instead of extract. The mean of three readings per sample was used, and TCC was expressed in milligrams of D-glucose equivalents (GE)/g of dry extract.

### 2.5. TLC Profiling

Adopting the methodology of [14], TLC profiling of extracts from the rhizome of *A. helferiana* and the bark of *S. fasciculata* was carried out. The particle-free extract solution in methanol was used for the TLC profiling. The solution band was applied to the silica gel F_254_ plates using a microcapillary tube. Initially, the extract-loaded plates were exposed to a hot air blow and then developed in a saturated glass beaker containing solvents (chloroform, methanol, and water) in a 6 : 4 : 1 proportion. The developed plates were again faced with blow to air the solvent. Subsequently, it was visualized in a UV chamber at (1) 254 nm, (2) 365 nm, (3) 10% v/v FeCl_3_ spray/dry, (4) 10% v/v H_2_SO_4_ spray/heat, and (5) dipped in 500 *µ*M DPPH solution in methanol.

### 2.6. DPPH Scavenging Assay

Spectrophotometrically, extracts of *A. helferiana and S. fasciculata* were assessed for their potency in scavenging free radicals from DPPH by implementing the method outlined by [11, 15]. In brief, test extracts (1.5 ml) were treated with 1.5 ml of fresh DPPH methanolic solution (100 *µ*M) in a microtitter plate. The plate was gently shaken for 10 seconds to mix the content. Subsequently, after 30 min of dark incubation at room temperature, at 517 nm, the absorbance of DPPH^•^ (oxidised form) in the mixture was measured against the DPPH control (containing 1.5 ml of distilled water instead of extract) and blank (distilled water). Ascorbic acid (0.6125 *µ*g/ml–5 *µ*g/ml) was considered as a reference antioxidant. The mean absorbance of triplicates per sample was utilized to calculate percentage DPPH^•^ scavenging.

(2) $$\begin{array}{c} \%\text{ DPPH radical scavenging}=\left[\frac{\left(A_{0}-A_{1}\right)}{A_{0}}\right]\times 100, \end{array}$$

where the *A*_0_ arrow is the absorbance of the DPPH^•^ control and *A*_1_ arrow is the absorbance of the test sample or the reference sample.

The antioxidant potency of each extract sample and positive control (ascorbic acid) was expressed as an IC_50_ value (mean ± standard deviation), i.e., the concentration in *µ*g/ml that scavenged DPPH^•^ absorbance by 50%. The linear graph that plots the percentage of DPPH^•^ scavenging against extract concentration and ascorbic acid was utilized to calculate IC_50_.

### 2.7. Antibacterial Activity

Clinical isolates of *S. aureus*, *E. coli*, and *P. aeruginosa* were used to evaluate the antibacterial activity of extracts adopting the well diffusion method [16] with a slight change. Bacterial colonies were transferred to a sterile test tube containing normal saline, and a 0.5 McFarland was prepared by maintaining an optical density (OD) of 0.1 at 620 nm. The solution of Muller–Hinton agar was prepared using distilled water. After autoclaving, the agar was aseptically poured into the sterile Petri plate. After solidification of the culture medium, the entire surface of each MHA plate was swabbed with 0.5 McFarland standard bacterial inoculums. In each petri plate, five wells were created using 6 mm sterile tips. 20 *μ*l of molten MHA was dropped to seal the base of the wells. 100 *µ*l of extract was added at doses of 25, 50, and 100 mg/ml in sterile normal saline to the corresponding wells, and plates were incubated at 37°C. As a negative control, normal saline was used, and as a positive control, standard antibiotic meropenem (MP-10 *μ*g) were used. After 48 hours, the clear zone of inhibition around the wells was measured in millimeters (mm).

### 2.8. Statistical Analysis

Statistical analysis was performed using Microsoft Excel 2019 software. Each experiment was carried out in triplicate, and the data were presented as mean ± standard deviation. The TPC, TFC, TCC, and antioxidant activity (IC_50_) were determined by linear regression analysis.

## 3. Results

### 3.1. Qualitative Phytochemical Analysis

The phytochemical analysis of *A. helferiana* and *S. fasciculata*, shown in Table 1, revealed the presence of numerous bioactive chemical constituents such as phenols, tannins, flavonoids, saponins, terpenoids, proteins, and carbohydrates. Alkaloids and anthraquinones were not detected.

### 3.2. Extraction Yield

The highest yield (22.81% w/w) was obtained from the rhizomes of *A. helferiana*, while the lowest yield (5.33% w/w) was obtained from the barks of *S. fasciculata* (Table 2).

### 3.3. Quantitative Phytochemical Analysis

The quantitative estimation of phenols, flavonoids, and carbohydrates in the extracts of *A. helferiana* and *S. fasciculata* is shown in Table 2. The linear regression equations of standard gallic acid (*y* = 0.001*x* − 0.014, *R*^2^ = 0.994), quercetin (*y* = 0.017*x* − 0.266, *R*^2^ = 0.98), and D-glucose (*y* = 0.019*x* + 0.172, *R*^2^ = 0.94) were implemented to estimate TPC, TFC, and TCC, respectively. The *A. helferiana* extract revealed a maximum, TPC of 208.33 ± 12.96 mg GAE/g extract, and TCC of 564.16 ± 2.92 mg·GE/g extract. The extract of *S. fasciculata* revealed a maximum TFC of 30.35 ± 0.1 mg QE/g of extract.

### 3.4. TLC Profiling

**Figure 2:**
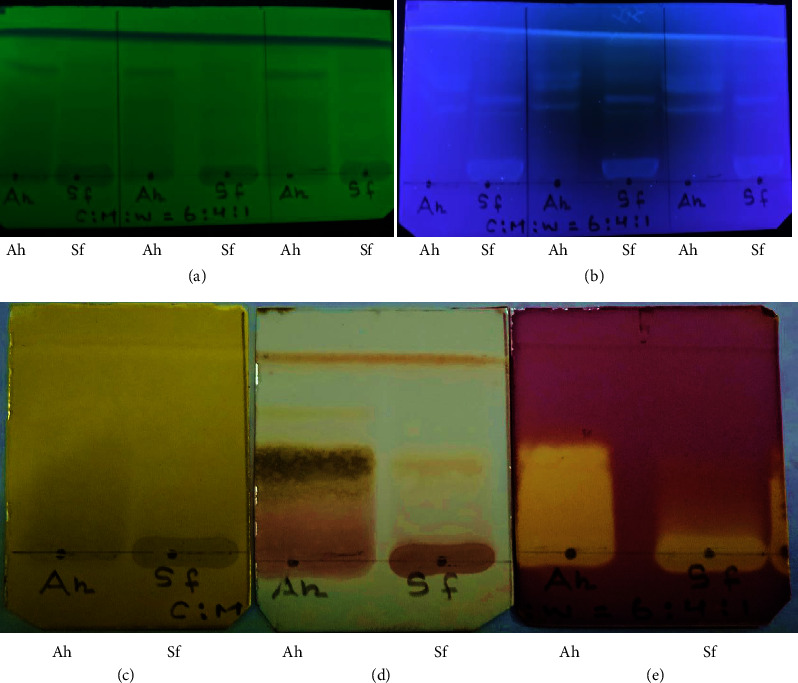
Thin layer chromatography (TLC) profiles of *A. helferiana* (Ah) and *S. fasciculata* (Sf) extracts using silica gel F_254_ as stationary phase and CHCl_3_ : MeOH : H_2_O = 6 : 4 : 1 v/v as the development solvent; observed under (a) UV-254 nm; (b) UV-365 nm; (c) using 10% FeCl_3_ spray reagent and dried; (d) using 10% H_2_SO_4_ spray reagent and heated; (e) dipped in 500 *µ*M DPPH solution.

The TLC profiles of extracts of *A. helferiana and S. fasciculata* are shown in Figure 2.

### 3.5. Antioxidant Activities

**Figure 3:**
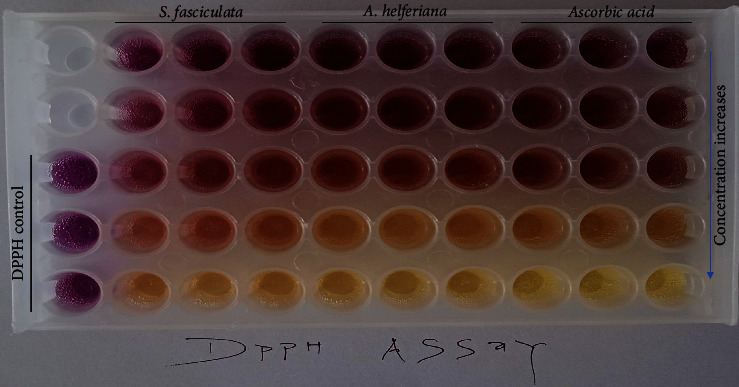
Photograph of the DPPH radical scavenging activity of *A. helferiana*, *S. fasciculata*, and ascorbic acid.

The antioxidant abilities of the extracts of *A. helferiana*, *S. fasciculata* extracts, and standard ascorbic acid against DPPH free radicals are shown in Table 3 and Figure 3. The extract of *A. helferiana* possesses strong DPPH free radical scavenging activity with an IC_50_ of 25.09 *µ*g/ml, whereas *S. fasciculata* and standard ascorbic acid possess an IC_50_ value of 31.07 *µ*g/ml and 2.79 *µ*g/ml, respectively.

### 3.6. Antibacterial Activity

Table 4 summarizes the antibacterial activity of the extract of *A. helferiana* and *S. fasciculata* against *S. aureus*, *P. aeruginosa*, and *E. coli*. Among the extracts tested against the selected bacteria, only *A. helferiana* rhizome extract exhibited potent antibacterial activity against *E. coli* with a zone of inhibition ranging from 15 to 20 mm.

## 4. Discussion

Plants have been used for their remedial properties since ancient times, and their secondary metabolites have long inspired researchers in the quest for novel chemical compounds with bioactive properties such as antioxidant, antiviral, anticancer, antibacterial, and anti-inflammatory activities [17–19]. Approximately 2000 plants are reported to be used for medicinal purposes in Nepal [1, 2]. Based on these facts, *A. helferiana* and *S. fasciculata* used in this study were selected in compliance with their ethnomedicinal uses in Nepal [3, 7].

70% MeOH was used for extraction due to its ability to partition a wide range of polarities and soluble phenolic compounds such as phenolic glycosides, flavonoids, phenolic acids, lignans, xanthones, coumarins, iridoids, chalcones, and terpenoids [9, 10, 14, 20]. The high yield of the *A. helferiana* rhizome extract could be due to the high content of polar and 70% methanol-soluble components (polyphenols, flavones, and carbohydrates) in the *A. helferiana* rhizome. As a result, 22% of the extraction yield is currently obtained from the red rhizome of *A. helferiana*, which is higher than the yield obtained from the local cultivar of *A. helferiana* with the yellow rhizome [3, 4].

Phytochemical analysis indicated that extracts of the rhizome of *A. helferiana* and the bark of *S. fasciculata* were potential sources of bioactive chemicals (Table 1). The current study on 70% MeOH extracts from the *A. helferiana* rhizome revealed higher TPC and TFC than those reported by previous studies carried out in the MeOH extract of *A. helferiana* rhizome [3]. Since 70% MeOH is more polar than MeOH [21], the higher TPC and TFC in 70% MeOH extract could be due to the partition of highly polar and water soluble compounds such as saponins, glycosides, and tannins [3, 21]. However, this study was the first to analyze the presence of phytochemicals and quantify (phenols, flavonoids, and carbohydrates) in the bark of *S. fasciculata* extracts.

The presence of several compounds in *A. helferiana* and *S. fasciculata* was confirmed by separation of several bands in chromatogram on visualized under UV at 254 and 365 nm (Figure 2). After 10% sulfuric acid spray/heat, yellow, black, and reddish-brown on the chromatogram of *A. helferiana* suggested the presence of flavonoids, carbohydrates, and terpenoids (including saponins), respectively [14]. Ferric chloride (FeCl_3_) is commonly used as a spray reagent to detect the phenolic compounds [14]. The chromatogram of *S. fasciculata* on FeCl_3_ spray showed spots at the bottom suggesting the presence of phenolic compounds. On dip in DPPH, a yellow band on the violet or purple background of the chromatogram revealed the presence of antioxidants in *A. helferiana* and *S. fasciculata* [14, 22, 23].

Plant-derived flavonoids and phenolic compounds have raised therapeutic interest due to their various medicinal properties, such as antioxidant, antiaging, and anti-inflammatory [15, 24]. Since the presence of flavonoids, phenolic compounds, and antioxidants in the extract of *A. helferiana* rhizomes and *S. fasciculata* barks was confirmed by present phytochemical analysis, the TPC, TFC, and TLC profiling. It seems necessary to estimate the antioxidant potency of the extracts. Therefore, we performed a DPPH radical scavenging assay to evaluate the antioxidant potency of the extracts.

Oxidative damage caused by free radicals is the pathophysiological basis for a wide array of disease conditions such as diabetes, hypertension, neurodegenerative disorders, coronary heart disease, and hepatotoxicity [11]. Antioxidants scavenge DPPH radicals by hydrogen atom transfer (HAT) or single electron transfer (SET), which ultimately leads to a change in color from purple to yellow after reduction [25, 26]. In this study, the 70% MeOH extract from red rhizomes of *A. helferiana* possessed dose-dependent free radical scavenging activity (Figure 3) with IC_50_ values of 25.9 *µ*g/ml, which is more potent than the yellow rhizomes of *A. helferiana* reported by [3]. However, the bark of *S. fasciculata* was studied for the first time and revealed potent antioxidant activity with an IC_50_ of 31.07 *µ*g/ml. The potent antioxidant activity could be due to phenolic, flavonoid, and terpenoid compounds [15, 24, 26, 27].

Globally, the emergence and spread of antibiotic resistance, as well as the evolution of new bacterial strains, has become a great concern to the human health [18, 28, 29]. Therefore, effective treatment of infectious diseases entails that the research and development of novel drugs from potent medicinal plants is necessary. The extract of *A. helferiana* exhibited the dose-dependent antibacterial activities against *E. coli*, similar to that of standard meropenem. Previously, Lamichhane et al. reported the angiopteroside from the yellow cultivar of *A. helferiana* [4]. Likewise, the authors in [30, 31] revealed significant antibacterial activity of angiopteroside against *E. coli*. Therefore, the potent antibacterial activity of the red cultivar of *A. helferiana* might be due to the angiopteroside. Additionally, the antibacterial activity might be contributed by phytoconstituents such as saponins [32], phenolic compounds [33], flavonoids [19], and terpenoids [34]. Several research studies have suggested that the phytoconstituents such as terpenoids, phenolic compounds, and flavonoids bind via hydrogen bonding with proteins and enzymes of bacterial cells, causing the disruption of cell membranes [35], inhibition of cell wall synthesis, and inhibition of enzymes necessary for amino acid synthesis [34], which leads to bacterial death. Interestingly, standard meropenem was found to be resistant with minimal zone of inhibition against *P. aeruginosa*. Similarly, both extracts were also found to be resistant against *S. aureus* and *P. aeruginosa*. This could be due to upregulation of hydrolytic beta lactamase, efflux pump, and porin mutations and downregulation of penicillin-binding proteins and influx passage of antibacterial agents into the bacterial cells [35–37]. Provided it acquires further isolation and experimental validation for antibacterial candidate development.

## 5. Conclusions

The current study confirmed the potent antioxidant activity of red rhizomes of *A. helferiana* and the barks of *S. fasciculata,* including the phytoconstituents such as such as phenols, flavonoids, and terpenoids. Additionally, *A. helferiana* rhizomes exhibited antibacterial activity against *E. coli.* Therefore, these plant extracts may serve as a source for novel lead structures in the development of antioxidant and antibacterial drugs to protect humans from several diseases related to oxidative stress and bacterial infections.

## Figures and Tables

**Table 1:** Qualitative phytochemical analysis of *A. helferiana* and *S. fasciculata* extracts.

Phytochemicals	Types of test	Crude liquid extract	
		*A. helferiana*	*S. fasciculata*
Phenols	(1) FeCl_3_ tests	+	+

Flavonoids	(1) Alkaline reagent tests	−	−
	(2) Shinoda tests	+	+
	(3) Lead acetate tests	+	−

Tannins	(1) FeCl_3_ tests	+	+
	(2) Lead acetate tests	+	−
	(3) Lime water tests	+	+
	(4) Gelatin tests	+	+

Saponins	(1) Foam tests	+	+

Alkaloid	(1) Mayer's reagent tests	−	−
	(2) Wagner reagent tests	−	−
	(3) Hager's reagent tests	—	—

Terpenoids and steroids	(1) Salkowski tests	+	+

Carbohydrates	(1) Fehling tests	+	+
	(2) Benedict tests	+	+

Protein/amino acids	(1) Ninhydrin tests	+	—

Anthraquinones	(1) Borntrager's tests	—	—

(+) indicates presence; (−) indicates absence.

**Table 2:** Extraction yield and total phenolic, flavonoid, and carbohydrate content of extracts of *A. helferiana* and *S. fasciculata*.

Extracts	Yield (%, w/w)	TPC (mg GAE/g extract)	TFC (mg QE/g extract)	TCC (mg GE/g extract)
*A. helferiana*	22.81	208.33 ± 12.96	26.15 ± 0.06	564.16 ± 2.92
*S. fasciculata*	5.336	66.66 ± 2.85	30.35 ± 0.10	137.68 ± 6.29

Extraction yield was expressed as % yield = (weight of dry extract/initial weight of dry sample) × 100. TPC: total phenolic content. TFC: total flavonoid content. TCC: total carbohydrate content. GAE: gallic acid equivalent. QE: quercetin equivalent. GE: D-glucose equivalent. The TPC, TFC, and TCC values are the mean ± standard deviation of triplicate experiments.

**Table 3:** DPPH scavenging activity of ascorbic acid, *A. helferiana*, and *S. fasciculata* extracts.

Samples	Conc (*µ*g/ml)	DPPH scavenging assay		
		Percentage scavenging	Linear regression equation, *R*^2^	IC_50_ (*µ*g/ml)
		(Mean ± SD)		
*A. helferiana* extract	50	90.594 ± 0.07	*Y* = 1.700*X* + 5.97, 0.997	25.9
	25	48.305 ± 0.09		
	12.5	29.777 ± 0.40		
	6.25	16.433 ± 0.43		
	3.125	9.501 ± 1.61		

*S. fasciculata* extract	50	76.194 ± 1.70	*Y* = 1.351*X* + 8.02, 0.998	31.07
	25	40.258 ± 6.13		
	12.5	25.586 ± 2.85		
	6.25	16.625 ± 1.72		
	3.125	12.396 ± 0.49		

Ascorbic acid^*∗*^	5	88.10 ± 11.06	*Y* = 17.58*X* + 0.88, 0.997	2.79
	2.5	47.1333 ± 0.451		
	1.25	20.720 ± 0.495		
	0.6125	12.163 ± 1.57		

DPPH: 2,2-Diphenyl-1-picrylhydrazyl. IC_50_: concentration required to scavenge 50% of free radicals in the sample solution. ^*∗*^Used as a standard antioxidant. Percent scavenging values are expressed as mean ± standard deviation.

**Table 4:** Antibacterial activity of extracts of *A. helferiana* and *S. fasciculata* using the agar-well diffusion method.

Bacteria	ZOI of extracts in millimeters		
	Conc (mg/ml)	*A. helferiana*	*S. fasciculata*
*S. aureus*	100	0	0
	50	0	0
	25	0	0
	0	0	0
	MP	25	25

*E. coli*	100	20	0
	50	15	0
	25	10	0
	0	0	0
	MP	20	25

*P. aeruginosa*	100	0	0
	50	0	0
	25	0	0
	0	0	0
	MP	8	8

*S. aureus*: *Staphylococcus aureus*. *E. coli*: *Escherichia coli*. *P. aeruginosa*: *Pseudomonas aeruginosa*. ZOI: zone of inhibition. Conc: concentration of extracts. 0 mg/ml: negative control. MP 10 *µ*g: positive control meropenem disc.

## Data Availability

Data used to support the findings of this study are included in this article.
